# The Itinerary of Autophagosomes: From Peripheral Formation to Kiss-and-Run Fusion with Lysosomes

**DOI:** 10.1111/j.1600-0854.2008.00701.x

**Published:** 2008-01-30

**Authors:** Luca Jahreiss, Fiona M Menzies, David C Rubinsztein

**Affiliations:** Department of Medical Genetics, Cambridge Institute for Medical Research, University of Cambridge Wellcome/MRC Building, Addenbrooke's Hospital, Hills Road, Cambridge CB2 0XY, UK

**Keywords:** autophagosome, autophagy, dynein, fusion, lysosome

## Abstract

Macroautophagy, a constitutive process in higher eukaryotic cells, mediates degradation of many long-lived proteins and organelles. The actual events occurring during the process in the dynamic system of a living cell have never been thoroughly investigated. We aimed to develop a live-cell assay in which to follow the complete itinerary of an autophagosome. Our experiments show that autophagosomes are formed randomly in peripheral regions of the cell. They then move bidirectionally along microtubules, accumulating at the microtubule-organizing centre, in a similar way to lysosomes. Their centripetal movement is dependent on the motor protein dynein and is important for their fusion with lysosomes. Initially, autophagosomes dock on to lysosomes, independent of lysosomal acidification. Two kinds of fusion then occur: complete fusions, creating a hybrid organelle, or more often kiss-and-run fusions, i.e. transfer of some content while still maintaining two separate vesicles. Surprisingly, the autophagolysosomal compartment seems to be more long lived than expected. Our study documents many aspects of autophagosome behaviour, adding to our understanding of the mechanism and control of autophagy. Indeed, although the formation of autophagosomes is completely different from any other vesicular structures, their later itinerary appears to be very similar to those of other trafficking pathways.

Macroautophagy (which we will refer to as autophagy) involves the formation of double-membrane vesicles around a portion of cytosol to form autophagosomes. These eventually fuse with lysosomes, where their contents are degraded. This process is conserved from yeast to man and plays key roles in the degradation of many long-lived cytosolic proteins and organelles. In this way, autophagy can buffer against starvation. However, autophagy also clears aggregate-prone intracellular cytosolic proteins that cause neurodegenerative diseases (e.g. Huntington's disease) and a range of pathogens (e.g. *Mycobacterium tuberculosis*) and is involved in antigen presentation and various cancers (1).

The understanding of mammalian autophagy has been greatly facilitated by pioneering studies from Ohsumi, Klionsky and others working in yeast as many of the yeast genes critical for the process have mammalian orthologues. However, there are numerous key differences between mammalian and yeast systems. For instance, in yeast, there is only one specific site of autophagosome formation, the pre-autophagosomal structure (PAS) next to the vacuole, while in mammalian cells, multiple sites of origin can be observed at the same time (2). An ever increasing number of parts of the molecular machinery of mammalian autophagy are being identified. For instance, the yeast Atg8 orthologue light chain 3 (LC3) appears to play a role in the expansion and completion of autophagosome formation. Indeed, LC3 is the only known protein that specifically associates with autophagosomes (in both inner and outer membranes) and autophagolysosomes and not with other vesicular structures (3). However, there are additional homologues of Atg8 (e.g. Golgi-associated ATPase enhancer of 16 kDa (GATE-16)) that associate with other structures, but haven't been directly implicated in autophagy. After fusion with lysosomes, LC3 can be localized either on the outside of the autophagolysosome membrane or be inside the vesicle. Green fluorescent protein (GFP)–LC3 on the outside of the autophagolysosome appears to be very rapidly removed, while that inside is subject to proteolytic degradation, and the GFP signal appears to persist if lysosomal proteolysis is inhibited or if pH is elevated (4).

Despite the current knowledge about the autophagy machinery, there are many aspects that are unclear. Much of our understanding of the itinerary of autophagosomes is derived from light and electron microscopy of fixed cells. This has led to concepts of initial autophagic vacuoles or autophagosomes, degradative autophagic vacuoles or autolysosomes (5) and amphisomes [vesicles resulting from fusions between (late) endosomes and autophagosomes] (6). However, the events occurring during the life cycle of an autophagosome and their regulation are only poorly understood. For instance, it is unclear where autophagosomes originate from, how they move and how they fuse with lysosome/late endosome compartments.

The machinery involved in autophagosome–lysosome fusion is well understood in yeast. Although some parts of the molecular machinery have been elucidated in mammalian cells (7–9) and suggest a similar process as in yeast, many aspects of autophagosome–lysosome fusion in mammalian cells remain mysterious. The actual nature of fusion and all the components involved remain to be investigated. Importantly, in yeast, autophagosomes form at the PAS, which is close to the vacuole, while in mammalian cells, there are multiple sites of autophagosome formation, which may be at some distance from lysosomes. This may result in a need for specific transport systems for autophagosomes in mammalian cells.

In this study, we have used a combination of live-cell imaging and fixed cell studies to allow us to investigate many aspects of the itinerary of an autophagosome. We demonstrate that autophagosomes form randomly throughout the cytoplasm. Once formed, autophagosomes need to reach lysosomes, enriched perinuclearly around the microtubule-organizing centre (MTOC) (10), in order to fuse with them and to degrade their contents. Microtubules seem to be essential for autophagosome movement (11,12); here, we show that autophagosomes move bidirectionally along microtubules and that their movement towards the nucleus is mediated by the motor protein dynein.

Our live-cell imaging studies show that autophagosome–lysosome fusion is similar to endosome–lysosome fusion, where kiss-and-run, complete fusions or fusion mediated through tubules can all be observed (13). Additionally, we demonstrate that docking and fusion appear to be two separately regulated events. As a whole, our study documents many aspects of autophagosome behaviour, adding to our understanding of the mechanism and control of autophagy. Indeed, although the formation of autophagosomes is completely different from any other vesicular structures, their later itinerary appears to be very similar to those of other trafficking pathways.

## Results

### Autophagosomes are formed randomly throughout the cytoplasm

In order to follow the whole itinerary of an autophagosome, the autophagosome-specific marker microtubule-associated protein 1 LC3 tagged to enhanced green fluorescent protein (EGFP) was expressed in living normal rat kidney (NRK) cells. Normal rat kidney cells were chosen for the majority of the experiments because they show a sufficient number of autophagosomes per cell in the basal unstarved state to allow efficient real-time data capture and at the same time are quite flat (thickness of approximately 2 μm), making it easier to follow dynamic processes within a single focal plane with a confocal microscope. Before embarking on studies using transfected fluorescently tagged LC3 constructs to mark autophagosomes for microscopical analyses, we confirmed that both GFP–LC3 and mCherry–LC3 were specifically labelling autophagosomes by demonstrating that the great majority of cells lose these vesicular structures when treated with 3-methyladenine, an inhibitor of autophagosome formation (data not shown). Furthermore, all fluorescently labelled LC3 structures were <1 μm in diameter, and no large aggregates were observed.

**Figure 1:**
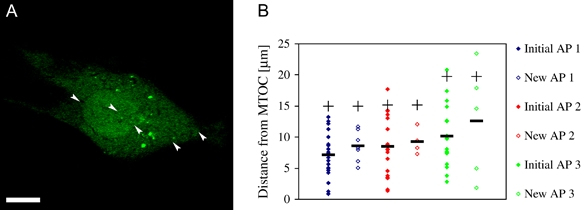
Autophagosomes are formed randomly throughout the cytosol upon serum starvation A) Location of initial autophagosomes (GFP–LC3-positive vesicles) and of newly appearing autophagosomes (see arrowheads for location of their appearance) during a 5-min video. B) Distance of initial and newly appearing autophagosomes from the MTOC of three different cells after 30- to 120-min starvation in serum-free CO_2_-independent medium (black bar indicates mean distance of autophagosomes from the MTOC, and plus sign indicates mean distance from the MTOC to the cell periphery). AP, autophagosome. Scale bar represents 10 μm.

First, we compared the locations of newly appearing autophagosomes, which were induced by serum starvation, with those of autophagosomes already present at the beginning of the observation. Serum starvation rapidly increased the number of autophagosomes (data not shown), as has previously been reported (14). In these starved cells, pre-existing autophagosomes were found throughout the cytosol with a somewhat higher density near the nucleus, whereas new autophagosomes were generally formed further towards the periphery with no specific localization (Figure 1), as has also been reported in other cell lines (9).

### A high proportion of autophagosomes are actually autophagolysosomes

**Figure 2:**
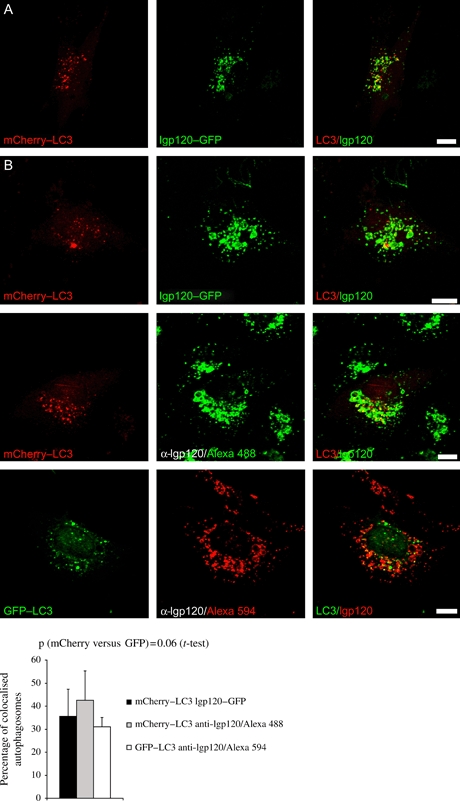
A high proportion of autophagosomes are actually autophagolysosomes A) Cells transfected with mCherry–LC3 and lgp120–GFP show a high degree of colocalization. B) Colocalization of mCherry–LC3 with lgp120 is somewhat higher than colocalization of GFP–LC3 with lgp120. Scale bars represent 10 μm.

We then investigated the end-point of the itinerary of an autophagosome, i.e. the fusion with a lysosome. It has previously been reported that GFP–LC3 fluorescence is lost rapidly upon autophagosome–lysosome fusion (15), because of both degradation and the low pH in the lysosome, as GFP itself has a pKa of about 6. Therefore, we used LC3 tagged with the novel red fluorescent protein mCherry (16), which has the advantage of a low pKa (<4.5). This construct indeed enabled us to visualize potential autophagolysosomes when cells were cotransfected with lgp120 [also known as lysosome-associated membrane glycoprotein 1, a glycoprotein mainly localized to the limiting membranes of lysosomes and late endosomes (17)] fused to GFP at its C-terminal cytosolic end. Thereby, a high fraction of LC3-positive vesicles were also lgp120 positive (Figure 2A). All vesicles positive for lgp120–GFP could be stained with a specific antibody against lgp120 (data not shown), and the majority of lgp120-positive vesicles were also acidic as determined by Lysotracker staining (data not shown). When the colocalization of GFP–LC3 with lgp120 was directly compared with the colocalization of mCherry–LC3 with lgp120 by using an antibody against lgp120, a high degree of colocalization could still be observed, although it was lower than that for mCherry–LC3 (Figure 2B). The higher colocalization of mCherry–LC3 with lgp120, compared with GFP–LC3, was consistent with recently published data comparing monomeric red fluorescent protein–LC3 and GFP–LC3 (4). In both cases, there was more colocalization of the tagged LC3 with the lower pKa, concordant with the data suggesting that most LC3 is inside autophagolysosomes, where it is sensitive to pH and proteolysis (4). The high proportion of LC3 vesicles positive for lgp120 was not restricted to NRK cells – it was also seen in Hela cells (Figure S1). These data suggest that the autophagolysosomal compartment, although inherently transient, is much more long lived than expected.

### LC3/lgp120 colocalization correlates with the level of autophagic activity

**Figure 3:**
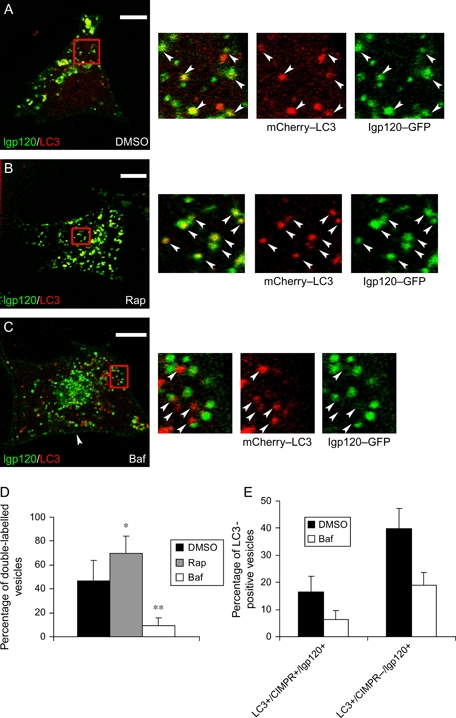
lgp120/LC3 colocalization correlates with level of autophagic activity A) Dimethyl sulphoxide (DMSO)-treated control with some colocalization (see magnification and arrowheads). B) Twenty-four hours rapamycin (Rap) treated with high level of colocalization (see magnification and arrowheads). C) Twenty-four hours bafilomycin (Baf) treated with low colocalization (see magnification and arrowheads). D) Quantification of percentage of double-labelled vesicles (mean of three experiments of four to five cells each, mean standard deviations; *t*-test: p (DMSO, Rap) = 0.008, p (DMSO, Baf) = 0.001). E) Quantification of colocalization of cells transfected with mCherry–LC3 and lgp120–GFP and stained for CIMPR. Cells were treated with 400 nm bafilomycin for 24 h (20 cells each, bars represent means with standard deviations, p (LC3+/CIMPR+/lgp120+) = 6 × 10^−12^, p (LC3+/CIMPR−/lgp120+) = 1 × 10^−7^). Scale bars represent 10 μm.

In order to test how meaningful this colocalization was in relation to autophagic activity, cells were treated with known enhancers and inhibitors of autophagy. Similar to serum starvation, treatment with the enhancer rapamycin increased the number of LC3-positive vesicles (data not shown) and increased endogenous LC3-II levels (Figure S4A) in NRK cells. Interestingly, rapamycin treatment also led to a significant increase in the proportion of double-labelled vesicles (Figure 3B,D). Blocking of autophagosome–lysosome fusion with bafilomycin (a proton pump inhibitor that inhibits late endosome and lysosome acidification), however, led to almost no double labelling at all (Figure 3C,D). The results of this assay correlate well with the known effects of these drugs on autophagy (18,19).

To test the effects of bafilomycin further, cells were additionally labelled with an antibody against the cation-independent mannose-6-phosphate receptor (CIMPR). This transmembrane protein is normally localized to the Golgi network and late endosomes, but is excluded from lysosomes, and has been previously been shown to localize to a subset of autophagosomes (20). This triple labelling enabled us to differentiate between amphisomes (LC3+/CIMPR+/lgp120+) and autophagolysosomes (LC3+/CIMPR−/lgp120+). Bafilomycin treatment decreased fusion of LC3-positive vesicles with both late endosomes and lysosomes (Figure 3E).

### Autophagosomes engage in different types of homotypic and heterotypic fusions

**Figure 4:**
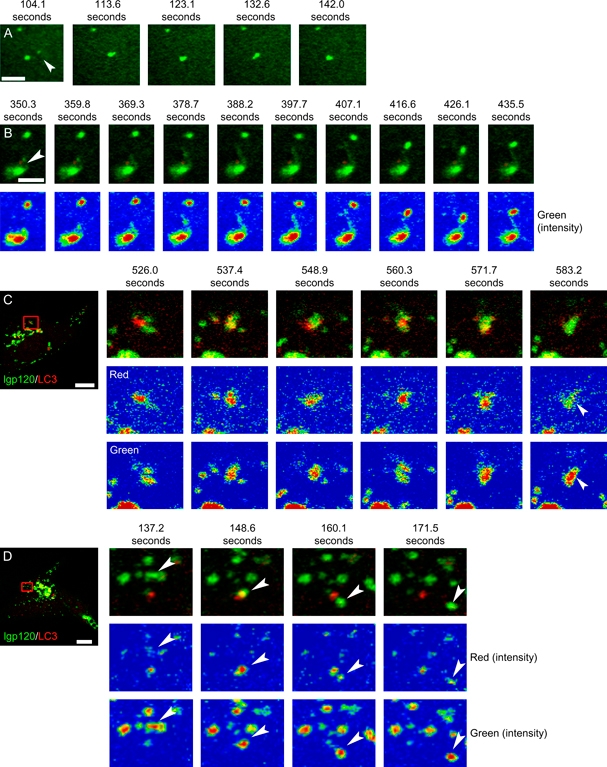
Autophagosomes engage in different types of both homotypic and heterotypic fusions A) Homotypic fusion between two GFP–LC3-positive autophagosomes (arrowhead). Scale bar represents 3 μm. B) Homotypic fusion through membrane protrusion (arrowhead). Scale bar represents 3 μm. C) Complete autophagosome–lysosome fusion resulting in colocalization of mCherry–LC3 and lgp120–GFP in the hybrid organelle (arrowheads). For better visualization of double labelling, images were split into two separate channels (red and green) while using false colour for a better measure of intensity (blue < green < yellow < red). When observing an autophagosome interacting with a lysosome, membrane content exchange would be visible as an increase in intensity in the red channel at the location of the lysosome and vice versa. Scale bar represents 10 μm. D) Kiss-and-run fusion event resulting in some mCherry–LC3 inside lgp120–GFP-positive vesicle (arrowheads). Scale bar represents 10 μm.

We investigated whether autophagolysosomes were generated by formation of a hybrid organelle by complete fusion of an autophagosome and a lysosome. When we analyzed GFP–LC3-transfected cells by live-cell imaging, homotypic fusion between two LC3-positive vesicles could sometimes be observed. Fusion was either complete (Figure 4A), i.e. fusion of two autophagosomes to form a new one, or through membrane protrusions (Figure 4B).

When both mCherry–LC3 and lgp120–GFP were transfected into cells, fusion events (as assayed by membrane content exchange) could be observed in about 23% of untreated cells (*n* = 26 cells, Figure 4C,D). Moreover, when cells were imaged shortly (30–100 min) after addition of rapamycin, these events occurred more frequently (33% of cells, *n* = 9 cells).

Two different types of fusion were observed. Sometimes (approximately 31% of fusions, *n* = 56 cells), the autophagosomes and late endosomes/lysosomes fused completely, resulting in a completely double-labelled hybrid vesicle (Figure 4C). However, more often (approximately 62% of fusions, *n* = 56 cells), the fusion seemed to be of the ‘kiss-and-run’ type. The sequence of events was the following: first, an lgp120–GFP-positive lysosome with no (or only a low amount of) mCherry–LC3 started interacting with an autophagosome. This led to an increase in the intensity of mCherry fluorescence in the lysosome. When they eventually separated, the lysosome retained the transferred mCherry–LC3 (Figure 4D). Rarely (7% of fusions, *n* = 56 cells), the same kind of fusion could also be observed in the opposite direction (Figure S2).

To further investigate whether the fusions observed involved late endosomes or lysosomes, the lysosomal lumen was specifically labelled by loading the cells with Oregon Green 488 dextran, as previously described (13). Kiss-and-run fusions between LC3-positive vesicles and dextran-loaded lysosomes could then also be observed (Figure S3). This led to a high level of double-labelled vesicles.

Thus, ‘kiss-and-run’ fusions seem to be most common type of fusion between autophagosomes and lysosomes. Moreover, the membrane content exchange seems to be unidirectional in that content is usually transferred only from autophagosomes to lysosomes. The transfer can be explained as the LC3 localized to the inner leaflet of the autophagosome being transferred into the lysosome, while the one in the outer leaflet is rapidly lost (e.g. by delipidation by Atg4) (4).

### Docking and fusion are two independent steps in the maturation of the autophagosome

**Figure 5:**
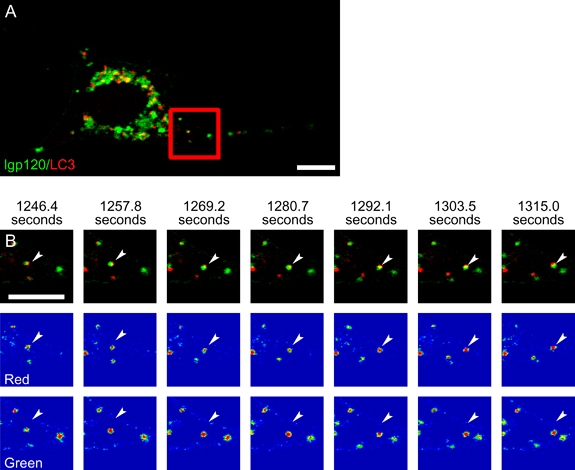
Docking and fusion are two independent steps in the maturation of the autophagosome A) Low LC3/lgp120 colocalization in cell treated with bafilomycin for 24 h. B) Enlargement of area in (A); autophagosome docked onto lysosome (arrowheads) with common movement, but no fusion (see separate intensities and Figure 4). Scale bars represent 10 μm. These data are representative of five independent experiments.

Treatment of the cells with vacuolar H+-adenosine triphosphatase (ATPase) inhibitor bafilomycin for 24 h clearly inhibited fusion between autophagosomes and lysosomes (Figures 3C,D and 5A), so that two distinct populations (LC3+/lgp120− or LC3−/lgp120+) of vesicles could be observed.

However, close interactions of autophagosomes with lysosomes without any obvious membrane content exchange still occurred (Figure 5B), suggesting that acidification of the lysosome is not a requirement for the docking of an autophagosome. Thus, docking and fusion are two mechanistically distinct steps in autophagosome–lysosome fusion.

### Autophagosomes move bidirectionally towards and away from the nucleus dependent on microtubules and associated motor proteins

**Figure 6:**
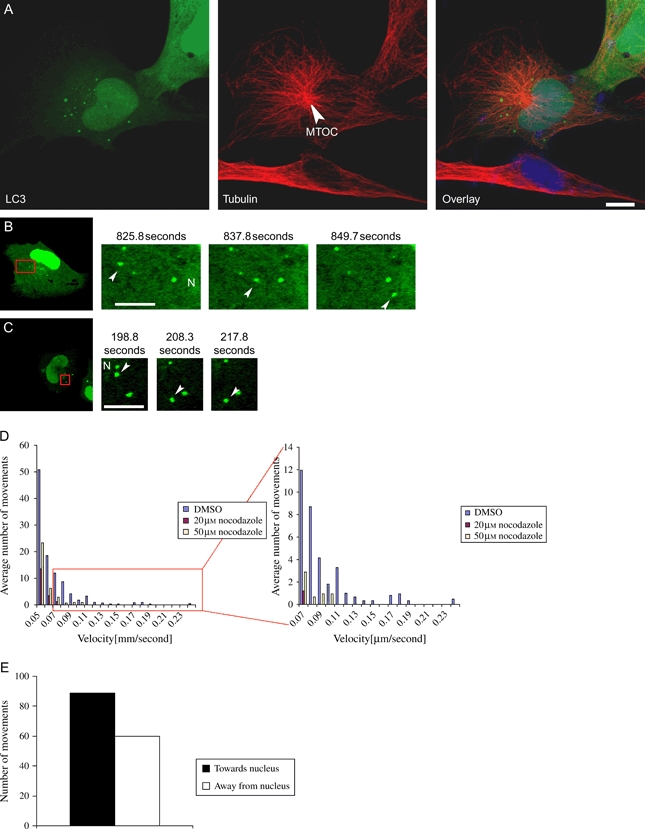
Autophagosomes move bidirectionally towards and away from the nucleus dependent on microtubules A) Autophagosomes are distributed with a bias towards the MTOC. Scale bar represents 10 μm. B) Fast autophagosome movement towards the nucleus (N) (arrowheads). Scale bar represents 5 μm. C) Fast autophagosome movement away from the nucleus (N) (arrowheads). Scale bar represents 5 μm. D) Fast movements with a velocity above 0.1 μm/second are abolished by microtubule depolymerization by nocodazole. E) Microtubule-dependent movements (i.e. velocity ≥ 0.1 μm/second) are biased towards the nucleus [p = 0.02 (chi-squared test), cumulative result from six cells, 10 vesicles each].

Autophagosomes are formed randomly throughout the cytoplasm (Figure 1) and fuse with lysosomes (Figure 4) in order to degrade their contents. Because the majority of lysosomes are localized perinuclearly (Figure 2) around the MTOC (data not shown), the autophagosomes need to be transported there, most likely along microtubules. When GFP–LC3 was overexpressed in unstarved NRK cells, autophagosomes were distributed with a bias towards the MTOC and were not randomly distributed (Figure 6A), similar to lysosomes (data not shown). This is compatible with our observations above that many LC3-positive structures are also lgp120 positive. Additionally, the LC3-positive vesicles colocalized quite well with microtubules (Figure 6A), suggesting a role for microtubule-dependent transport systems in autophagosome movement.

This suggested that autophagosomes are delivered to the vicinity of the lysosomes near the MTOC in a dynein/microtubule-dependent fashion through productive movements biased in direction towards the nucleus. In order to test this, we first assessed the movement of autophagosomes in live NRK cells expressing GFP–LC3, thus labelling both autophagosomes and autophagolysosomes. When the vesicles were tracked and a velocity histogram was plotted, the resulting distribution was clearly positively skewed (Figure 6D). For a large part, especially close to the nucleus, the vesicles seemed quite immobile or only moved short distances. When autophagosomes did move over longer distances, the movements were both centripetal (Figure 6B) and centrifugal (Figure 6C), but only very rarely tangential, as others have also reported (11). To test whether these fast movements were actually dependent on microtubules, they were depolymerized by a short treatment (30 min) with nocodazole (data not shown). This abolished all movement above a velocity of 0.1 μm/second (Figure 6D). Therefore, this velocity was chosen as the threshold for microtubule-dependent movements. Importantly, there was a significant bias for microtubule-dependent movements towards the nucleus over movements away from the nucleus [chi-squared test (H_0_: no bias): p = 0.02; Figure 6E). Together with interactions and fusion events with lysosomes near the nucleus working as an additional sink for autophagosome transport, this explains their aforementioned prominent perinuclear accumulation. Altogether, these results suggest that autophagosomes move along microtubules in a bidirectional manner.

**Figure 7:**
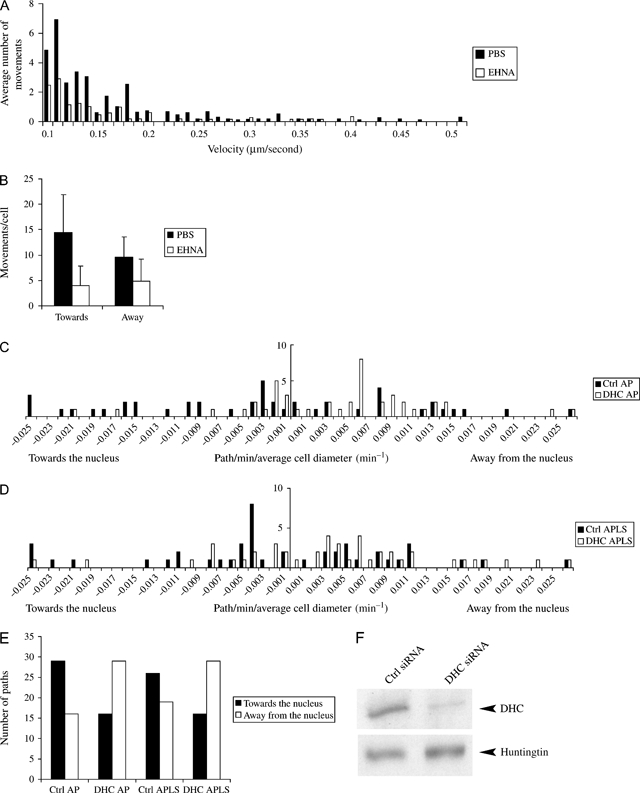
Dynein mediates the movement of autophagosomes and autophagolysosomes towards the nucleus A) Reduction in fast movements upon dynein inhibition (*n* = 6; Mann–Whitney *U*-test: p (EHNA, PBS) = 0.02). Six cells were analyzed in detail. In each cell, we determined the distribution of single movements in between each frame (11.5 seconds per frame) for 10 autophagosomes (any LC3-positive vesicle) per cell. Autophagosomes were tracked for as long as they were visible, and all their movements were recorded. As mentioned in the text, the same autophagosome can show both fast movements and periods of immobility during the observation period. The averaged distributions of movements per frame of fast movements (as characterized in Figure 6) over the six cells are shown. B) Data from (A) were analyzed as follows. For each frame, we determined if the direction of the movement was towards or away from the nucleus. The numbers were recorded for each cell, and the means and standard deviations for the six cells analyzed are shown. Dynein inhibition by EHNA decreases movement towards nucleus more strongly [*n* = 6; *t*-test: p (towards) = 0.02, p (away) = 0.08)]. Towards the nucleus (Towards), away from the nucleus (Away). C and D) Dynein knockdown decreases paths of autophagosomes (AP, LC3+/lgp120−) (C) and autophagolysosomes (APLS, LC3+/lgp120+) (D) towards the nucleus. The path of an autophagosome was defined as the distance covered from the beginning of tracking till the end, with the associated direction. For standardization between different vesicles and cells, these values were then divided by the duration of tracking and the mean diameter of the cell (the mean diameter of an ellipse approximating the cell shape). E) Dynein knockdown significantly changes the bias in direction of paths [defined in (D) above] from towards the nucleus to away from the nucleus (pooled data from 45 vesicles [three experiments, three cells each, five vesicles each), p (AP) = 0.00005 (chi-squared test), p (APLS) = 0.003 (chi-squared test)]. F) Efficient knockdown of dynein heavy chain [because of the high molecular weight of DHC (532 kD), endogenous wild-type huntingtin (343 kD) was used as a loading control].

We tested if autophagosome movements towards the MTOC were mediated by dynein (the motor protein that typically moves cargoes along microtubules in this direction) by treating cells with the adenine analogue erythro-9-[3-(2-hydroxynonyl)]adenine (EHNA), a dynein ATPase activity inhibitor (21). EHNA preferentially affected movements of GFP–LC3 vesicles directed towards the nucleus (Figure 7A,B). Similar to what has been described for reducing dynein activity in other systems (22), EHNA also had a smaller secondary effect on movements away from the nucleus.

To further test the involvement of dynein in autophagosome movement, dynein heavy chain was knocked down by RNA interference (Figure 7F). Because, as previously mentioned, a large fraction of LC3-positive vesicles might actually represent autophagolysosomes (Figure 2A), we cotransfected mCherry–LC3 and lgp120–GFP to be able to distinguish between the two types of vesicles, which might move differently. Additionally, because autophagosomes moved both towards and away from the nucleus, we decided to focus on the productive movement by analyzing their path, i.e. their overall movement between the starting and the end-point of tracking. As expected, dynein knockdown led to a decrease in the number of paths towards the nucleus and a concomitant increase in the number of paths away from the nucleus (Figure 7C,D,E). Both autophagosomes and autophagolysosomes were similarly affected. Thus, the movement towards the nucleus of both autophagosomes and autophagolysosomes is mediated by dynein.

**Figure 8:**
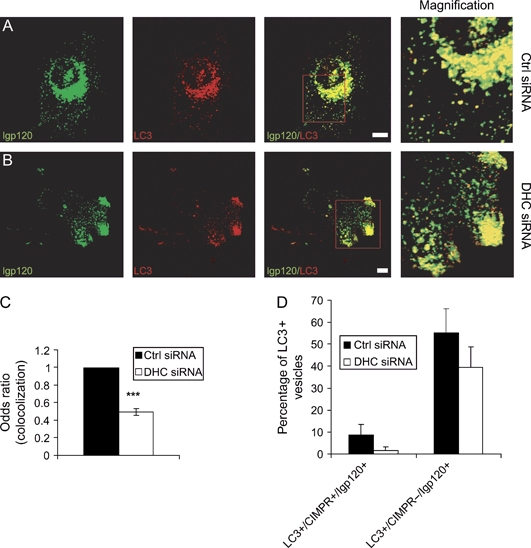
Dynein knockdown redistributes autophagosomes towards the periphery and decreases autophagosome–lysosome fusion A) Mostly perinuclear localization of autophagosomes/autophagolysosomes in control cells. B) Redistribution of autophagosomes/autophagolysosomes towards the periphery with a concomitant reduction in autophagosome–lysosome fusion (see magnification). C) Quantification of reduction in colocalization of LC3 with lgp120 [three experiments with 20 cells each, p < 0.0001 (odds ratio)]. D) Quantification of colocalization of cells transfected with control (Ctrl) or DHC siRNA, mCherry–LC3 and lgp120–GFP and stained for CIMPR (20 cells each, bars represent means with standard deviations, p (LC3+/CIMPR+/lgp120+) = 4 × 10^−6^, p (LC3+/CIMPR−/lgp120+) = 3 × 10^−5^). Scale bars represent 10 μm.

Consistent with the above data, dynein knockdown led to a redistribution of LC3-positive vesicles towards the cell periphery and membrane extensions, compared with control cells, where they were mainly localized around the nucleus (Figure 8A,B). Concomitantly, we observed a clear reduction in the fraction of autophagolysosomes (Figure 8C), suggesting decreased fusion. Additionally, similar to the effects of bafilomycin treatment (Figure 3E), dynein knockdown led to decreased fusion of LC3-positive vesicles with late endosomes and lysosomes (Figure 8D) [The higher occurrence of autophagolysosomes compared with Figure 3E was because of the longer expression of mCherry–LC3 (72 h compared with 24 h)]. Finally, similar to what we have previously shown (23), dynein knockdown increased the levels of LC3-II (data not shown) and decreased the clearance of the autophagic substrate α-synuclein A53T (data not shown), underscoring the importance of dynein-mediated movement for autophagic activity.

### Rapamycin treatment does not affect autophagosome dynamics

As we have shown in this study and a previous study (23), autophagosome movement is important for autophagic activity. Therefore, we tested whether the induction of autophagy by rapamycin treatment affected autophagosome dynamics. However, rapamycin treatment had no significant effect on the half-life of GFP–LC3-positive vesicles (Figure S4B), which we determined to be approximately 13 min, in line with what has previously been reported (12,24).

Moreover, autophagy induction had no effect on the velocities of movements of autophagosomes or autophagolysosomes (Figure S4C,D).

Thus, the increase in autophagic clearance upon autophagy induction seems to be largely because of the increase in autophagosome formation, leading to increase cargo uptake and delivery.

## Discussion

In conclusion, our data suggest that autophagosomes are formed randomly in more peripheral regions of the cell, in most cases far away from the nucleus. Therefore, we can exclude formation at the MTOC or any perinuclearly localized organelles, such as the Golgi.

Autophagosomes then move bidirectionally along microtubules towards and away from the MTOC, where they accumulate in a similar way to lysosomes because of a bias towards centripetal movements. These centripetal movements are dependent on the motor protein dynein and are rate limiting for their eventual fusion with perinuclearly located lysosomes. These data can explain why there is defective autophagosome–lysosome fusion associated with decreased dynein activity (23). Interestingly, LC3 binds to microtubules directly *in vitro* by electrostatic interactions (25) through its N-terminal domain (26), suggesting that it might be directly involved in autophagosome movement. Moreover, it has been reported that LC3 directly interacts with the dynein complex and that microinjection of anti-LC3 antibody inhibits autophagosome movement (27). Unfortunately, we could not test this further as almost no GFP-positive autophagosomes could be observed when an N-terminal deletion mutant of LC3 tagged to GFP was overexpressed in NRK or HeLa cells, suggesting that, similar to yeast (28), this domain is important for autophagosome formation or targeting of LC3 to the membrane (our unpublished observation).

Initially, autophagosomes dock on to lysosomes, a process that is independent of lysosomal acidification. From our colocalization experiments, we can conclude that a high fraction of LC3-positive vesicles are autophagolysosomes, which had previously been suggested (29). Our observation suggests the important conclusion that not all LC3-positive vesicles are identical and that many such structures are autophagolysosomes (as opposed to autophagosomes).

As previously reported (6), autophagosomes also fuse with endosomes, forming so-called amphisomes (Figures 3E and 8D). However, it remains unclear whether those fusions with endosomes are a prerequisite for later fusion with lysosomes (6). It is certainly a straightforward explanation; however, it is hard to test as inhibiting endosome–autophagosome fusion will likely have indirect effects on lysosomal biogenesis. When cells were treated with bafilomycin A1 or with dynein small interfering RNA (siRNA), we observed decreased fusion of LC3-positive vesicles with late endosomes and lysosomes. These treatments themselves block endosome maturation (30). However, the effects of dynein of centripetal autophagosome movement are likely to be a major contributor to decreased fusion with late endosomes and lysosomes.

We observed two types of autophagosome–lysosome fusions: complete fusions, where a hybrid organelle is formed, or, more often, kiss-and-run fusions, i.e. transfer of some content while still maintaining two separate vesicles. It is possible that autophagosomal content may be delivered to lysosomes by multiple events or that the delivery of lysosomal hydrolases to autophagosomes may create what appear to be degradative autophagosomes. Irrespective of these possibilities, the presence of both complete fusion and kiss-and-run events suggests different routes for autophagosome trafficking. It is interesting to speculate that these different routes may influence the efficiency of disposal of autophagic contents.

## Materials and Methods

### Constructs, antibodies and siRNA

We are grateful to T. Yoshimori for GFP–LC3, M. Mizuguchi for human LC3B, P. Luzio for GFP–lgp120 and R. Tsien for mCherry.

The following antibodies were used: polyclonal anti-CIMPR (1:1000, a gift from M. Seaman), polyclonal anti-dynein heavy chain (1:200, R-325; Santa Cruz), monoclonal anti-huntingtin (1:1000, MAB2166; Chemicon), polyclonal anti-LC3 (1:2000; Novus), mouse anti-rat lgp120 (1:250, a gift from P. Luzio), monoclonal anti-tubulin [1:500 immunocytochemistry (ICC), 1:5000 western blotting (WB); Sigma], anti-mouse Alexa 488 (1:500; Invitrogen), anti-mouse Alexa 594 (1:500; Invitrogen) and anti-rabbit Alexa 633 (1:1000; Invitrogen).

For the knockdown experiments, rat dynein heavy chain siRNA was used (on-target plus SMARTpool L-080024-01-0010; Dharmacon).

### Cloning of mCherry-hLC3B construct

Human light chain 3B (LC3B) was subcloned from pGEX-6P-1 (26) into pcDNA3 (Invitrogen) using *Bam* HI and *Eco* RI (both New England Biolabs (NEB)). mCherry (16) was amplified by polymerase chain reaction with the following primers: 5′-TA CCG AGC TCG GTA CCC GCC ACC AT-3′ and 3′-G CTG TAC AAG CAA GGA TCC TGC-5′. The resulting fragments were purified (Qiagen gel extraction kit), digested with *Kpn* I and *Eco* RI (both NEB) and subcloned in frame into the 5′ end of hLC3B pcDNA3.

### Cell culture

Normal rat kidney cells were grown in DMEM (Sigma) supplemented with 10% FBS (Invitrogen), 100 U/mL penicillin/streptomycin (Invitrogen) and 2 mml-glutamine (Invitrogen) at 37°C and 5% CO_2_. For live-cell imaging, cells were seeded on 42-mm glass cover slips (PeCon GmbH) at a density of approximately 1.5 × 10^5^ cells per cover slip or alternatively on 22 × 22-mm cover slips in a 6-well plate at a density of 1.5 × 10^5^ cells per cover slip for later fixation. For drug treatments, cells were treated for 24 h with 0.2 μg/mL rapamycin (Sigma), 400 nm bafilomycin A1 (Upstate) or 50 μm EHNA (Sigma). For starvation, cells were transferred to CO_2_-independent medium (Invitrogen) for 30–120 min.

### Transfection

Cells were transfected 24 h after seeding with 1.5 μg GFP–LC3 or 1 μg mCherry–LC3 and 0.5 μg lgp120–GFP in 6 μL Lipofectamine per cover slip for 4 h in DMEM. They were then washed once in full medium and cultured in full medium for the times indicated, unless stated otherwise in the figure legends (see Figure 1 legend). For siRNA experiments, cells were transfected with 20 nm siRNA, 0.5 μg mCherry–LC3, 0.25 μg lgp120–GFP and 5 μL Lipofectamine 2000 in Optimem (Invitrogen) for 4 h. They were then washed once in full medium and cultured in full medium for 48 h. Finally, they were split, reseeded at 1.5 × 10^5^ cells per cover slip and cultured for another 24 h until imaging/fixation.

### Dextran loading

Right after 4 h of transfection, cells were loaded with 0.5 mg/mL lysine-fixable 10 kD Oregon Green 488 dextran (Molecular Probes) for 4 h at 37°C in full medium, followed by a chase of 20 h in dextran-free full medium, as previously described (13).

### Immunocytochemistry

For the tubulin staining, cover slips were washed once in ×1 PBS, fixed for 20 min in 4% paraformaldehyde, washed once in ×1 PBS, permeabilized in ×1 PBS 0.2% Triton-X-100 for 5 min and washed three times in ×1 PBS. Then, they were blocked in blocking buffer (10% FBS and 1% BSA in ×1 PBS) for 1 h. Primary antibody diluted in blocking buffer was added, and cover slips were incubated at 4°C overnight. The next day, cells were washed three times with ×1 PBS, incubated with the appropriate secondary antibody and washed again. Finally, they were mounted in Prolong Gold antifading solution (Invitrogen) containing 4’,6-diamidino-2-phenylindole (DAPI) (3 μg/mL; Sigma).

For the lgp120 staining, the procedure was the same as above, except that the cover slips were fixed 10 min in −20°C in methanol.

For the CIMPR staining, cells were fixed in 4% paraformaldehyde (PFA) and permeabilized in 40 μm digitonin. Primary and secondary antibodies were only incubated for 1 h each at room temperature in 3% BSA (in PBS).

### Western blotting

Cell pellets were lysed on ice in Laemmli buffer (62.5 mm Tris–HCl pH 6.8, 5% β-mercaptoethanol, 10% glycerol and 0.01% bromophenol blue) for 30 min in the presence of protease inhibitors (Roche Diagnostics). Samples were subjected to SDS–PAGE, and proteins were transferred to a polyvinylidene fluoride (PVDF) membrane (GE Healthcare). Blots were first probed with primary antibodies (see above). Then, they were probed with the appropriate anti-mouse or anti-rabbit immunoglobulin G-horseradish peroxidase (GE Healthcare) secondary antibody and visualized using a enhanced chemiluminescence detection kit (GE Healthcare).

### Live-cell imaging

Twenty-four hours after transfection, the cover slips were mounted in a perfusion, open and closed cultivation (POC) chamber (PeCon GmbH) in CO_2_-independent medium (Invitrogen), and the temperature was equilibrated at 37°C. The imaging was performed on a Carl Zeiss with a LSM 510 confocal attachment using a ×63 1.4 NA Plan Apochromat oil immersion lens. The POC cell chamber was heated to 37°C with a heated stage and Tempcontrol 37 device (Carl Zeiss). Laser lines at 488 nm (GFP-tagged constructs, Oregon Green 488 dextran) and 543 nm (mCherry–LC3) were used. Band pass (505–530 nm) and long pass (560 nm) filters were used to separate wavelengths. For the dynein heavy chain (DHC) siRNA experiments, the imaging was performed as above but with the microscope fitted with an XL-3 incubator (PeCon GmbH) and using a 594-nm laser line instead of 543 nm. Laser power was kept at a minimum to minimize photobleaching and photocytotoxicity. The detector pinholes were set to give a 0.9-μm optical slice to minimize the chances of vesicles going out of/coming into focus (as mentioned in the *Results*, NRK cells are approximately 2 μm in thickness). Scan rates varied from 1.9 to 3.9 seconds by using multitracking (line switching) with a line average of 2 and with a delay time of 7.5 seconds in between scans. Acquisition was performed using zeisslsm 510 software. Acquisition times varied from 5 to 20 min. The maximum total imaging time per cover slip was 90 min.

### Live-cell imaging analysis

Movies were analyzed in zeisslsmimagebrowser 3.5 for double labelling, interactions and fusions. Vesicle tracking was performed in imagej (http://rsb.info.nih.gov/ij/) using the *LSM reader* and *Manual tracking* plug-ins. Thereby, 10 vesicles (GFP–LC3 only) or five LC3+/lgp120− and five LC3+/lgp120+ vesicles (mCherry–LC3/lgp120–GFP), which could be tracked for at least 15 frames (approximately 2.5 min), were chosen at random for each cell. These vesicles were then tracked manually for as long as they were visible, while the program calculated velocities for each frame [i.e. distance divided by total time in between frames (scan time + delay time)]. The results were copied to ms excel, where all further calculations and analyses were performed. Directions relative to the nucleus were determined from the algebraic sign of differences of distances (i.e. towards the nucleus corresponds to a negative difference) from an arbitrary point in the densest part of the cell near the nucleus, where autophagosomes accumulated. At the end of each tracking, the directions of fast movements were then rechecked by eye. Because of the resolution limits of the light microscope and inherent errors of manually clicking on single pixels, we used double the mean pixel diagonal divided by the total time (i.e. 2√2 × 0.15 μm/9.5 seconds = 0.045 μm/second) as the lower threshold for velocity data.

The path of an autophagosome was defined as the distance covered from the beginning of tracking till the end, with the associated direction. For standardization between different vesicles and cells, these values were then divided by the duration of tracking and the mean diameter of the cell (the mean diameter of an ellipse approximating the cell shape).

### Colocalization analysis

Cover slips were blinded and 5 (dimethyl sulphoxide/Rap/Baf), 7 (GFP–LC3 versus mCherry–LC3) or 20 (DHC siRNA) cells were imaged on a Zeiss Axiovert 200M microscope with a LSM 510 confocal attachment using a ×63 1.4 NA Plan Apochromat oil immersion lens. Laser lines at 488 nm (lgp120–GFP), 543 nm (mCherry–LC3) and 633 nm (Alexa 633) were used.

These cells were then analyzed in zeiss lsm image browser 3.5 as follows: first, after switching off all other channels, all mCherry–LC3-positive vesicles were counted and marked. Then the GFP channel was switched back on, and the number of colocalized vesicles was counted. For the triple-labelling experiments, the colocalization with CIMPR was finally quantified for the single or double-labelled vesicles from the analysis above. From these values, the fraction of double/triple-labelled vesicles was determined, i.e. the percentage of LC3-positive vesicles labelled with another/both other markers was calculated.

### Statistical analysis

Student's *t*-test and chi-squared test were performed in ms excel and Mann–Whitney *U*-test and log-rank test in graphpad prism 4. Odds ratios were determined by unconditional logistical regression analysis, using the general log-linear analysis option of spss 9 software (SPSS).

## Supplementary Materials

Figure S1 Other cell lines also show a high proportion of autophagolysosomes HeLa cells transfected with mCherry–LC3 and lgp120–GFP show a high degree of colocalization.

Figure S2 Rare transfer of lgp120–GFP into mCherry–LC3-positive vesicle Rare kiss-and-run fusion event resulting in some lgp120–GFP inside mCherry–LC3–positive vesicle (arrowheads). For better visualization of double labelling, images were split into two separate channels (red and green) while using false colour for a better measure of intensity (blue < green < yellow < red). When observing an autophagosome interacting with a lysosome, membrane content exchange would be visible as an increase in intensity in the red channel at the location of the lysosome and vice versa.

Figure S3 Kiss-and-run fusion with dextran-loaded lysosome Kissand-run fusion event between mCherry–LC3–positive autophagosome (red) and Oregon Green 488 dextran-loaded lysosome (green). In the middle panel of the image sequence, the lysosome (green, open arrowhead) can be observed docking onto the autophagosome (red), with a concomitant content exchange (filled arrowhead), leading to a double-labelled vesicle.

Figure S4 Autophagosome formation can be induced with rapamycin without an effect on autophagosome dynamics A) Twelve hours rapamycin treatment increases endogenous LC3–II levels in NRK cells. B) Twenty-four hours rapamycin treatment has no significant effect on the half-life of GFP–LC3–positive vesicles (mean and standard errors, three experiments of three cells each, log-rank test: p = 0.9). Cells were imaged for 16 min and then the times of disappearance of all GFP–LC3–positive vesicles visible at the start of the session were collected. From these data, the percentage of remaining vesicles was determined for each time-point. C and D) Twenty-four hours rapamycin treatment has no significant effect on fast autophagosome (AP, panel C) or autophagolysosome (APLS, panel D) movements (Figure 7) [five experiments, three cells each, five vesicles each; Mann–Whitney U-test: p (AP) = 0.1, p (APLS) = 0.6].
