# Supplementary figures and images for: The Itinerary of Autophagosomes: From Peripheral Formation to Kiss-and-Run Fusion with Lysosomes
Source: Traffic. 2008 Jan 30;9(4):574–87. doi: 10.1111/j.1600-0854.2008.00701.x (PMC2329914; doi:10.1111/j.1600-0854.2008.00701.x)

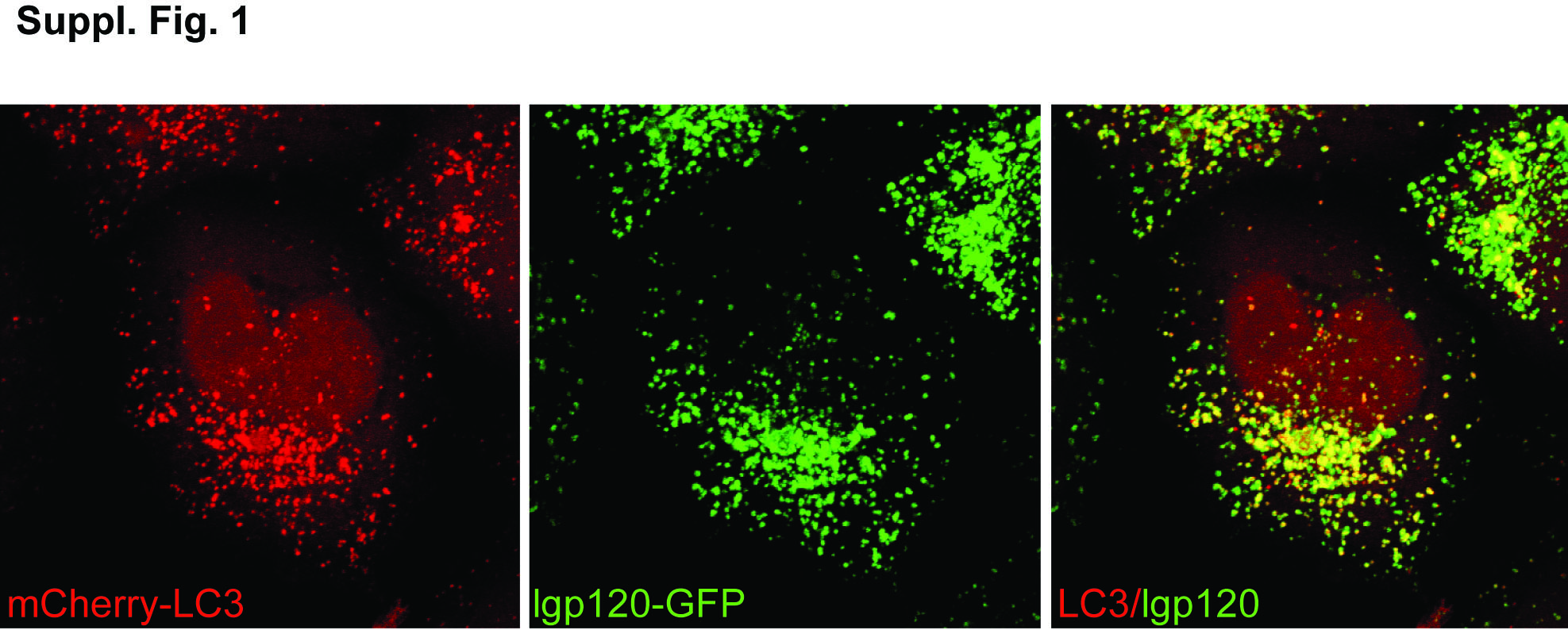

Supplement: Figure S1 — HeLa cells transfected with mCherry–LC3 and lgp120–GFP show a high degree of colocalization. [file tra0009-0574-SD1.jpg]

Suppl. Fig. 2

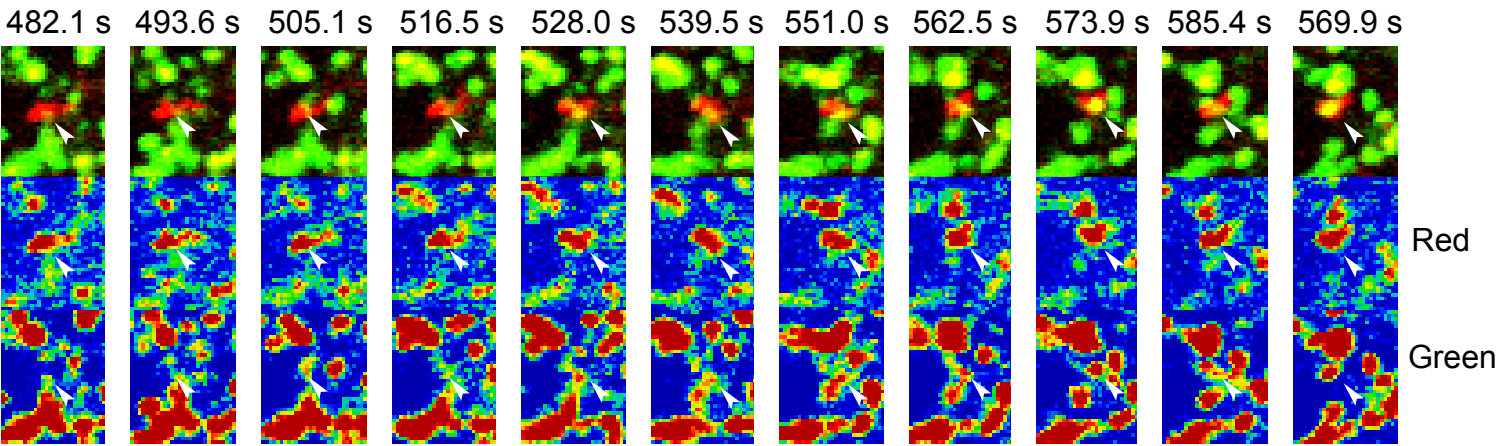

Supplement: Figure S2 — Rare kiss-and-run fusion event resulting in some lgp120–GFP inside mCherry–LC3–positive vesicle (arrowheads). For better visualization of double labelling, images were split into two separate channels (red and green) while using false colour for a better measure of intensity (blue < green < yellow < red). When observing an autophagosome interacting with a lysosome, membrane content exchange would be visible as an increase in intensity in the red channel at the location of the lysosome and vice versa. [file tra0009-0574-SD2.pdf]

Suppl. Fig. 3

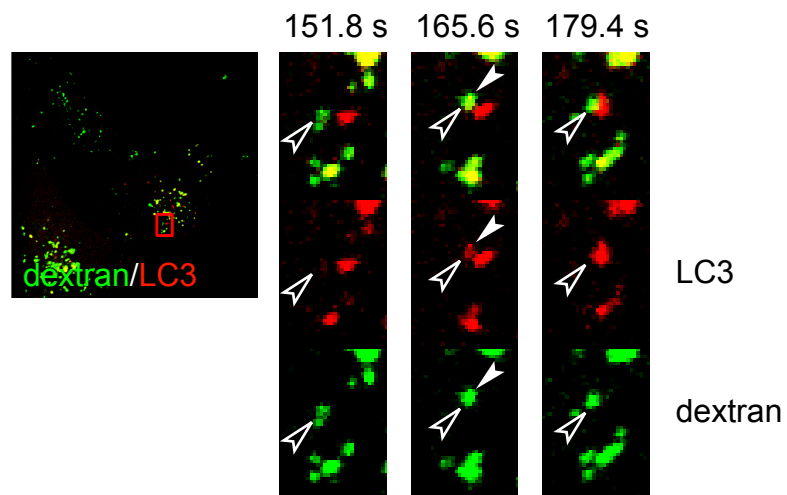

Supplement: Figure S3 — Kissand-run fusion event between mCherry–LC3–positive autophagosome (red) and Oregon Green 488 dextran-loaded lysosome (green). In the middle panel of the image sequence, the lysosome (green, open arrowhead) can be observed docking onto the autophagosome (red), with a concomitant content exchange (filled arrowhead), leading to a double-labelled vesicle. [file tra0009-0574-SD3.pdf]

Suppl. Fig. 4

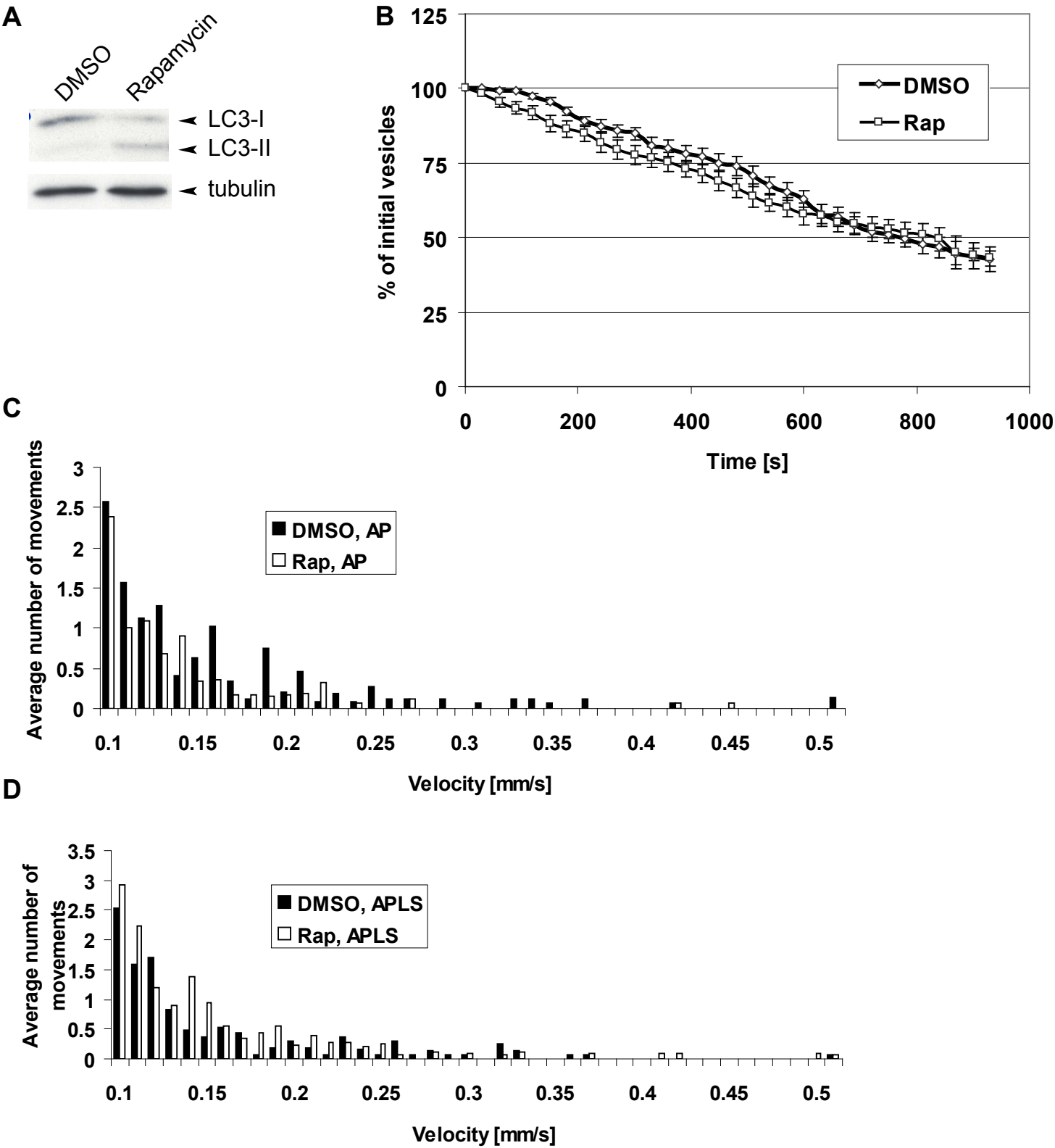

Supplement: Figure S4 — A) Twelve hours rapamycin treatment increases endogenous LC3–II levels in NRK cells. B) Twenty-four hours rapamycin treatment has no significant effect on the half-life of GFP–LC3–positive vesicles (mean and standard errors, three experiments of three cells each, log-rank test: p = 0.9). Cells were imaged for 16 min and then the times of disappearance of all GFP–LC3–positive vesicles visible at the start of the session were collected. From these data, the percentage of remaining vesicles was determined for each time-point. C and D) Twenty-four hours rapamycin treatment has no significant effect on fast autophagosome (AP, panel C) or autophagolysosome (APLS, panel D) movements (Figure 7) [five experiments, three cells each, five vesicles each; Mann–Whitney U-test: p (AP) = 0.1, p (APLS) = 0.6]. [file tra0009-0574-SD4.pdf]
